# The PPARβ/δ-AMPK Connection in the Treatment of Insulin Resistance

**DOI:** 10.3390/ijms22168555

**Published:** 2021-08-09

**Authors:** David Aguilar-Recarte, Xavier Palomer, Walter Wahli, Manuel Vázquez-Carrera

**Affiliations:** 1 Department of Pharmacology, Toxicology and Therapeutic Chemistry, Institute of Biomedicine of the University of Barcelona (IBUB), Faculty of Pharmacy and Food Sciences, University of Barcelona, Avinguda Joan XXIII 27-31, 08028 Barcelona, Spain; d.aguilarrecarte@gmail.com (D.A.-R.); xpalomer@ub.edu (X.P.); 2 Pediatric Research Institute-Hospital Sant Joan de Déu, 08950 Esplugues de Llobregat, Spain; 3 Spanish Biomedical Research Centre in Diabetes and Associated Metabolic Diseases (CIBERDEM)-Instituto de Salud Carlos III, 28029 Madrid, Spain; 4 Center for Integrative Genomics, University of Lausanne, CH-1015 Lausanne, Switzerland; walter.wahli@unil.ch; 5 Lee Kong Chian School of Medicine, Nanyang Technological University Singapore, Singapore 308232, Singapore; 6 ToxAlim (Research Center in Food Toxicology), INRAE, UMR1331, CEDEX, 31300 Toulouse, France

**Keywords:** PPARβ/δ, AMPK, GDF15, insulin resistance, type 2 diabetes mellitus

## Abstract

The current treatment options for type 2 diabetes mellitus do not adequately control the disease in many patients. Consequently, there is a need for new drugs to prevent and treat type 2 diabetes mellitus. Among the new potential pharmacological strategies, activators of peroxisome proliferator-activated receptor (PPAR)β/δ show promise. Remarkably, most of the antidiabetic effects of PPARβ/δ agonists involve AMP-activated protein kinase (AMPK) activation. This review summarizes the recent mechanistic insights into the antidiabetic effects of the PPARβ/δ-AMPK pathway, including the upregulation of glucose uptake, muscle remodeling, enhanced fatty acid oxidation, and autophagy, as well as the inhibition of endoplasmic reticulum stress and inflammation. A better understanding of the mechanisms underlying the effects resulting from the PPARβ/δ-AMPK pathway may provide the basis for the development of new therapies in the prevention and treatment of insulin resistance and type 2 diabetes mellitus.

## 1. Insulin Resistance: A Major Determinant of Type 2 Diabetes Mellitus

The prevalence of type 2 diabetes mellitus has reached global epidemic proportions and is one of the medical challenges of the 21st century [1]. Type 2 diabetes mellitus is defined by the presence of fasting hyperglycemia, which is responsible for the development of long-term complications, a decreased quality of life, and premature death [1]. It should be noted that abnormal glucose regulation may begin more than 10 years before the diagnosis of type 2 diabetes mellitus with the development of obesity-associated insulin resistance, which is defined as an impairment in the ability of insulin to maintain glucose homeostasis. However, at this early stage, subjects are asymptomatic, with glycemic values near normal levels because pancreatic islets usually respond by increasing insulin secretion to maintain normoglycemia in a process known as β cell compensation. Over time, β cell compensation for insulin resistance fails, resulting in fasting hyperglycemia and the establishment of type 2 diabetes mellitus [2]. As insulin resistance precedes and predicts type 2 diabetes mellitus [3], the development of new effective pharmacological approaches that prevent or delay its progression to type 2 diabetes mellitus relies on targeting the underlying pathological mechanisms. This is of paramount importance as current treatment options do not adequately control hyperglycemia or prevent the negative impact of type 2 diabetes mellitus in all patients. Among the new pharmacological strategies for treating obesity-induced insulin resistance and type 2 diabetes mellitus, Peroxisome Proliferator-Activated Receptor (PPAR)β/δ agonists show promise [4,5,6]. Ligands of this nuclear receptor have been reported to ameliorate insulin resistance and type 2 diabetes mellitus mainly through the activation of AMP-activated protein kinase (AMPK), a central regulator of multiple metabolic pathways. This review summarizes the recent mechanistic insights into how PPARβ/δ activates AMPK to ameliorate insulin resistance and type 2 diabetes mellitus.

## 2. Basic PPARβ/δ and AMPK Features

**Figure 1:**
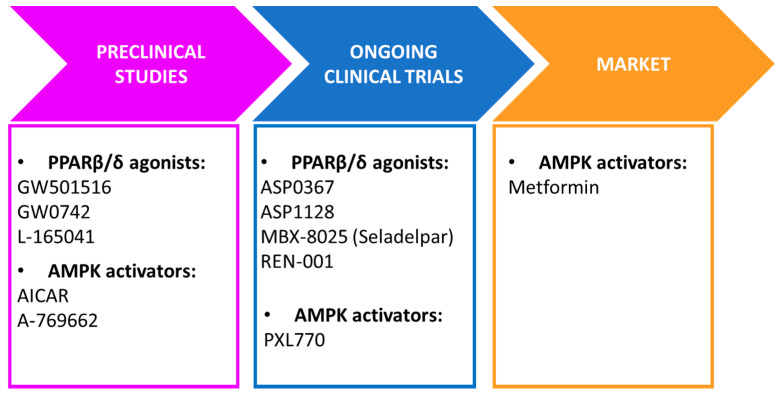
PPARβ/δ agonists and AMPK activators and current status in clinical pipeline. AICAR, 5-aminoimidazole-4-carboxamide ribonucleoside.

PPARs are members of the nuclear receptor superfamily of ligand-inducible transcription factors. The PPAR subfamily comprises three isotypes: PPARα (NR1C1: nuclear receptor subfamily 1, group C, member 1, according to the nomenclature agreed by the NC-IUPHAR Subcommittee on Nuclear Hormone Receptors), PPARβ/δ (NR1C2), and PPARγ (NR1C3) [4,5,6]. The PPARβ/δ isotype is ubiquitously expressed, but is most abundant in metabolically active tissues/cells, mainly those associated with fatty acid (FA) metabolism such as skeletal and cardiac muscle, hepatocytes, and adipocytes, and in macrophages. Ligand binding and activation of PPARβ/δ lead to its heterodimerization with its obligate dimerization partner retinoic acid receptor (RXR or NR2B). These heterodimers then bind to peroxisome proliferator response elements (PPREs) located in the promoters of their target genes to regulate their transcription. PPARβ/δ also regulates gene expression through DNA-independent mechanisms via crosstalk with other transcription factors [4,5,6]. Furthermore, it has been proposed that PKCα is a binding partner of PPARβ/δ, suggesting it as a mechanism through which the receptor may impact platelet reactivity [7]. Another example for a non-genomic effect of PPARβ/δ is the ligand-dependent interaction of the receptor with T-cell protein tyrosine phosphatase 45 (TCPTP45), which enhances insulin signaling [8]. In addition, the physiological activation status of PPARβ/δ depends on the presence of tissue-enriched specific ligands and the recruitment of coactivators or corepressors. Many of the target genes regulated by PPARβ/δ are involved in lipid and glucose metabolism, tissue repair, and inflammation [4,5,6]. The natural ligands of all PPAR isotypes are polyunsaturated and saturated FAs and their derivatives, but most of them show little receptor isotype selectivity. The development of several synthetic ligands with a high affinity and specificity for PPARβ/δ (GW501516, GW0742, and L-165041) has helped the understanding of the functions and pharmacology of this nuclear receptor [6] (Figure 1). Although no selective PPARβ/δ agonists have yet been approved for human use, several ongoing clinical trials are studying the efficacy and safety of several compounds selectively targeting this nuclear receptor: ASP0367 and ASP1128 (Mitobridge/Astellas Pharma, Cambridge, USA), MBX-8025 or Seladelpar (CymaBay Therapeutics, Neward, NJ, USA), and REN-001 (Reneo Pharmaceuticals, San Diego, CA, USA).

Over the last twenty years, many studies have robustly demonstrated that PPARβ/δ is crucial in regulating lipid metabolism and glucose homeostasis. Consequently, its activation is especially helpful in experimental models to prevent insulin resistance, type 2 diabetes mellitus, and associated metabolic disorders. Interestingly, many of the antidiabetic effects of the PPARβ/δ activators involve the activation of AMPK [6].

AMPK is a protein kinase that protects against insulin resistance and is activated by a low cellular energy status and glucose starvation [9]. These conditions, which activate AMPK, are signaled by the rise of the cellular AMP/ATP and ADP/ATP ratios. Once activated, AMPK triggers catabolic pathways that generate ATP and inhibits anabolic pathways that consume ATP. The heterotrimeric structure of AMPK comprises the α catalytic subunit and the regulatory β and γ subunits [9,10,11]. The binding of AMP to the γ subunit promotes AMPK activation through the phosphorylation of a conserved threonine (Thr172) residue within the α subunit via three complementary mechanisms: (1) phosphorylation by the upstream kinases liver kinase B1 (LKB1), Ca^2+^/calmodulin-dependent protein kinase kinase β (CaMKKβ), and transforming growth factor β-activated kinase 1 (TAK1); (2) inhibition of Thr172 dephosphorylation by protein phosphatases; and (3) allosteric activation. In addition to AMP, ADP also activates AMPK through mechanisms 1 and 2, while ATP inhibits these three mechanisms [9,10,11].

Given the importance of AMPK in lowering insulin resistance and associated metabolic disorders, many AMPK activators with different mechanisms of action have been developed. The most important AMPK activator is metformin, which is the most prescribed drug for type 2 diabetes mellitus treatment (Figure 1). However, its mechanism of action remains to be fully elucidated [12]. It has been reported that pharmacological metformin concentrations directly activate AMPK. By contrast, suprapharmacological metformin concentrations inhibit mitochondrial complex I, thereby reducing mitochondrial ATP production and increasing cellular AMP levels that subsequently activate AMPK [10,12]. A novel direct AMPK activator, PXL770 (Poxel), is being evaluated in an ongoing clinical trial (ClinicalTrials.gov 3 August 2021). In addition, many natural products, including resveratrol [13] and berberine [14], also indirectly activate AMPK by increasing cellular AMP levels. Another group of AMPK activators are AMP analogs, such as 5-aminoimidazole-4-carboxamide ribonucleoside (AICAR), which activate the γ subunit of AMPK [15]. A different group of ligands, exemplified by A-769662, includes synthetic direct activators that promote the allosteric activation of AMPK and the protection against Thr172 dephosphorylation [16,17]. Tetrahydrofolate analogs such as pemetrexed and methotrexate constitute another group of AMPK activators. These molecules inhibit the metabolism of ZMP, the phosphorylated form of AICAR, and promote its accumulation and subsequent activation of AMPK [18,19]. Finally, AMPK inhibitors are also useful in elucidating the effects mediated by this kinase. Compound C/dorsomorphin is an ATP-competitive AMPK inhibitor. However, this inhibitor is not specific for AMPK and shows AMPK-independent cellular effects [20]. More recently, a new direct inhibitor of AMPK has been characterized, SBI-0206965, with a 40-fold greater potency than compound C [21].

Once AMPK is activated, it phosphorylates key metabolic substrates and transcriptional regulators that affect many aspects of cellular metabolism, increasing glucose uptake, FA oxidation, mitochondrial oxidative capacity, and insulin sensitivity [22,23]. Interestingly, a high-fat diet (HFD) reduces AMPK phosphorylation levels in the skeletal muscle, liver, and other tissues, thereby indicating that restoration of the activity of this kinase can overcome metabolic alterations associated with the overconsumption of fat in animal models.

## 3. PPARβ/δ as a Major Regulator of Insulin Resistance through AMPK Activation

In the following sections of the review, we discuss studies that implicate AMPK activation in the antidiabetic effects of PPARβ/δ ligands in the main organs involved in insulin resistance.

### 3.1. Skeletal Muscle

**Figure 2:**
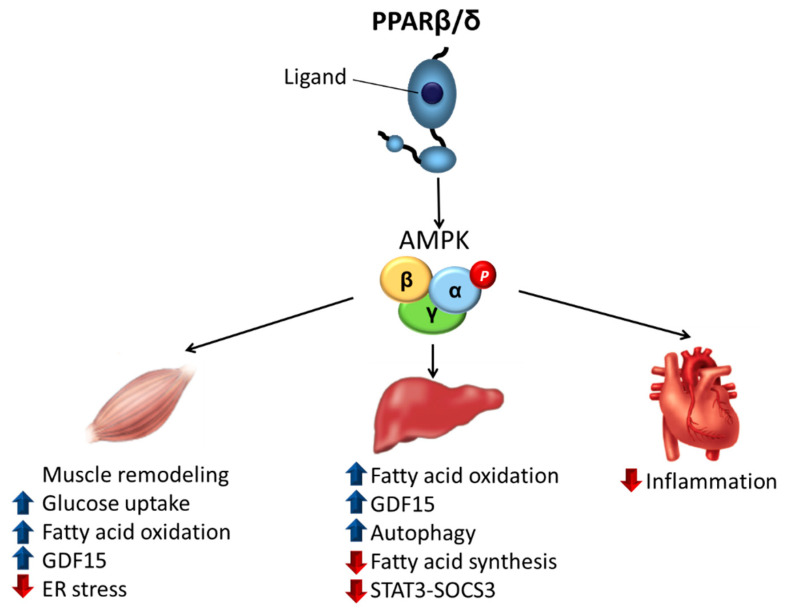
Antidiabetic effects of the PPARβ/δ-AMPK pathway in different organs. AMPK, AMP-activated protein kinase; ER, endoplasmic reticulum; GDF15, growth differentiation factor 15; PPARβ/δ: peroxisome proliferator-activated receptor β/δ; SOCS3: suppressor of cytokine signaling 3; STAT3: signal transducer and activator of transcription 3.

**Figure 3:**
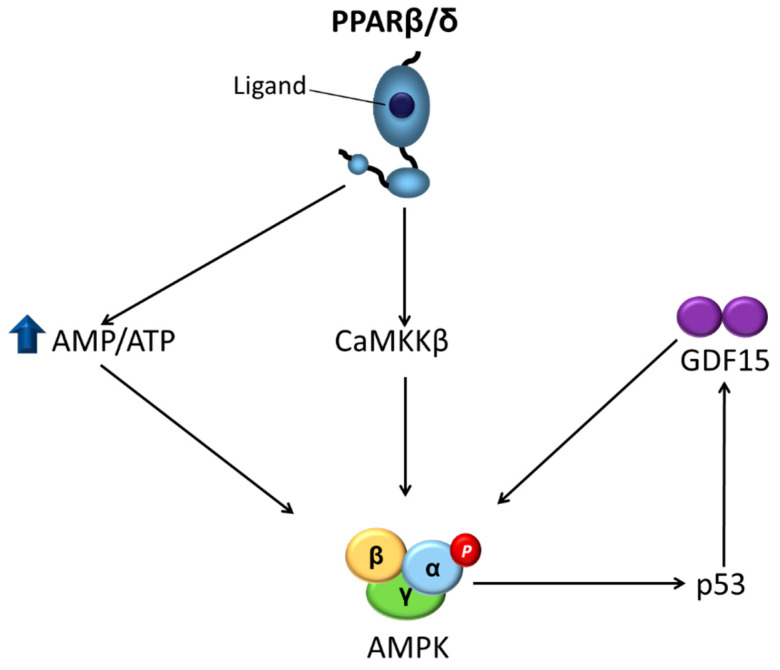
Mechanisms involved in the activation of AMPK by PPARβ/δ. AMPK is activated by PPARβ/δ through three mechanisms: (1) an increased AMP/ATP ratio; (2) an increased transcription of CaMKKβ; and (3) increased levels of GDF15 that sustain AMPK activation. AMPK, AMP-activated protein kinase; CaMKKβ, Ca^2+^/calmodulin-dependent protein kinase kinase-β; GDF15, growth differentiation factor 15; PPARβ/δ: peroxisome proliferator-activated receptor β/δ.

The primary site of insulin resistance in obesity and type 2 diabetes mellitus is the skeletal muscle, as it accounts for around 80% of insulin-stimulated glucose disposal [24,25,26]. Activation of AMPK in skeletal muscle by contraction (a process that results in a significant decrease in cellular ATP levels) or by activators of this kinase is associated with an insulin-independent mechanism that stimulates glucose transporter 4 (GLUT4) vesicle trafficking to the plasma membrane, resulting in elevated glucose transport into muscle, which lowers plasma glucose levels. This mechanism involves the phosphorylation by AMPK of tre-2/USP6, BUB2, cdc16 domain family member 1 (TBC1D1) and TBC1D4 (also known as Akt substrate of 160 kDa, AS160) [27], and phosphatidylinositol 3-phosphate 5-kinase [28]. Contrary to what was initially believed, a recent study suggested a role for AMPK in the regulation of insulin-stimulated glucose uptake [29]. The PPARβ/δ agonist GW501516 was reported to upregulate basal and insulin-stimulated glucose uptake in cultured primary human skeletal myotubes through AMPK activation [30], providing a role for AMPK in the antidiabetic effects of PPARβ/δ agonists (Figure 2). The authors of the study later reported that the activation of AMPK by GW501516 could be due to a reduction of the cellular energy status, as they observed an increase in the AMP/ATP ratio [31] (Figure 3). Moreover, transgenic mice with muscle-specific overexpression of PPARβ/δ show increased levels of mitochondrial enzymes and oxidative muscle fibers, which are more resistant to fatigue than glycolytic fibers, resulting in enhanced running endurance [32]. Notably, this overexpression of PPARβ/δ is accompanied by AMPK activation, with GW501516 and exercise training synergistically increasing oxidative myofibers and running endurance [33] (Figure 2). In the skeletal muscle of these mice, there is an interaction between PPARβ/δ and AMPK that is accompanied by more glycogen stores, increased levels of GLUT4, and an augmented capacity for mitochondrial pyruvate oxidation [34]. Thus, PPARβ/δ mimics the effects of endurance exercise training and GW501516 could be used as an exercise mimetic. In fact, this compound, sold under the name of Cardarine, has been misused for performance enhancement [35] and was entered into the list of prohibited substances in 2009 by the World Anti-Doping Agency [36]. This effect of PPARβ/δ was initially reported not to be associated with an increase in the mRNA levels of PPARγ co-activator 1α (PGC-1α) [32]. PGC-1α mediates mitochondrial biogenesis and its upregulation is associated with adaptation to endurance exercise through increased muscle mitochondrial numbers. However, later studies confirmed that PPARβ/δ does increase the protein levels of this transcriptional co-activator [37,38]. More recently, an elegant study revealed the mechanisms by which PPARβ/δ increased PGC-1α levels and activated AMPK in skeletal muscle during exercise [39]. PPARβ/δ increased PGC-1α protein levels via a post-transcriptional mechanism by protecting it from degradation through binding to PGC-1α and limiting its ubiquitination. PPARβ/δ also promoted the transcription of nuclear respiratory factor 1 (NRF-1), resulting in increases in the mitochondrial respiratory chain and in the transcription of CaMKKβ, ultimately leading to AMPK activation [38] (Figure 3). Overall, these findings showed that PPARβ/δ is essential for the maintenance and increase in mitochondrial enzymes, unveiling a new mechanism through which this nuclear receptor activates AMPK. This conclusion is supported by the phenotype of mice in which PPARβ/δ is selectively ablated in skeletal muscle myocytes. This somatic mutation causes a muscle fiber-type switching toward lower oxidative capacity that results in markedly reduced capacity to sustain running exercise, obesity, and type 2 diabetes mellitus [37].

In obesity, as the amount of visceral adipose tissue increases, so does the rate of lipolysis. This increases FA mobilization and raises the levels of circulating non-esterified FAs, which induce insulin resistance in skeletal muscle through activation of toll-like receptor (TLR)-dependent mechanisms or by promoting the accumulation of deleterious complex FA derivatives such as diacylglycerol (DAG) and ceramides. These pathways ultimately activate kinases (IκB kinase β, c-Jun N-terminal kinase 1, and protein kinase Cθ) that phosphorylate insulin receptor substrate 1 (IRS-1) on serine residues, attenuating the insulin signaling pathway [40]. The activation of PPARβ/δ in myotubes has been reported to transcriptionally upregulate the expression of target genes involved in FA β-oxidation such as pyruvate dehydrogenase kinase 4 (PDK4) and carnitine palmitoyltransferase-1β (CPT-1β). The increase in the expression of these genes promotes FA β-oxidation and reduces their availability to form complex lipids that induce insulin resistance [41] (Figure 2). CPT-1β, which catalyzes the rate-limiting step of mitochondrial FA oxidation, is inhibited by malonyl-CoA, a product of acetyl-CoA carboxylase (ACC) [22]. AMPK phosphorylates and inhibits ACC, thereby causing a decrease in intracellular malonyl-CoA levels, relieving CPT-1β inhibition and increasing FA oxidation. Therefore, PPARβ/δ activation in skeletal muscle increases mitochondrial FA oxidation by upregulating the expression of the target genes involved in this process as well as through increasing CPT-1β activity by phosphorylating AMPK.

In obese patients, the release of free FAs from visceral adipose tissue is also an important factor that triggers endoplasmic reticulum (ER) stress. This process induces insulin resistance by several mechanisms including the activation of inflammatory pathways, which activate the serine/threonine kinases that phosphorylate IRS-1 on serine residues [42]. PPARβ/δ ligands inhibit ER stress in skeletal muscle through a mechanism that seems to involve AMPK activation and the subsequent inhibition of extracellular signal-regulated kinase (ERK1/2) (Figure 2). In fact, AMPK activation protects against several deleterious processes by reducing ER stress [43,44,45,46]. Notably, there is inhibitory crosstalk between AMPK and ERK1/2 [47], with the inhibition of ERK1/2 promoting AMPK and Akt signaling and reversing ER stress-induced insulin resistance in skeletal muscle cells [48]. Therefore, PPARβ/δ ligands seem to require the activation of AMPK to inhibit ER stress, which strongly contributes to the antidiabetic effects of these compounds.

Recently, we reported that the metabolic effects caused by the pharmacological activation of PPARβ/δ may involve the stress-activated cytokine growth differentiation factor 15 (GDF15) [49]. This divergent member of the transforming growth factor β (TGFβ) superfamily [50] plays an important role in several biological processes, including the regulation of energy homeostasis [51]. In fact, overexpression of *Gdf15* in mice ameliorates glucose tolerance and insulin sensitivity and lowers body weight, although no difference in food intake was observed [52]. By contrast, administration of GDF15 to rodents reduces food intake and ameliorates glucose tolerance. Interestingly, a recent study reports that high pharmacological doses of GDF15 used in most studies reduce food intake, while physiological induction of endogenous circulating GDF15 levels does not affect it [53]. Although TGFβ receptors were initially reported to mediate the effects of GDF15, the presence of TGFβ contamination in recombinant GDF15 and the lack of a direct binding of GDF15 to known TGFβ receptors led to the search for the bona fide receptor of GDF15. Four independent groups reported in 2017 that GDF15 signals through the glial cell line-derived neurotrophic factor (GDNF)-like alpha-1 (glial cell-derived neurotrophic factor receptor alpha-like (GFRAL))/rearranged during transfection (RET) co-receptor complex [54,55,56,57]. The expression of GFRAL is limited to the central nervous system, specifically in the area postrema of the brainstem and parts of the nucleus of the solitary tract. Its activation by GDF15 in obesity improves glucose tolerance by reducing food intake. However, it has been reported that GDF15 also regulates metabolic parameters independently of changes in food intake [58], suggesting that GDF15 might also exert its effects via other receptors and peripheral mechanisms. We have reported recently that PPARβ/δ ligands increase GDF15 levels through an AMPK-p53-dependent mechanism [49]. Interestingly, the beneficial effects of the PPARβ/δ agonist GW501516 on glucose intolerance, FA oxidation, ER stress, inflammation, and AMPK activation in HFD-fed mice were abrogated by the injection of a GDF15-neutralizing antibody as well as in *Gdf15*^-/-^ mice. More importantly, these findings demonstrated that the increase in GDF15 caused by PPARβ/δ activation resulted in AMPK activation that did not require central effects, as these effects were observed in cultured myotubes and isolated muscle, suggesting the presence of autocrine/paracrine effects for GDF15 in skeletal muscle for which the mediating receptor remains to be identified (Figure 3). Although additional studies are needed to reject the potential involvement of GFRAL on the GDF15-mediated antidiabetic effects of PPARβ/δ agonists, as *Gfral* mRNA is absent in C2C12 cells [49,55] and skeletal muscle [49,59], the GDF15-mediated activation of AMPK in isolated skeletal muscle and cultured myotubes seems to exclude this receptor. The question that remains unanswered is the identity of the new potential receptor responsible for the autocrine/paracrine effects of GDF15 in skeletal muscle. Future studies should shed light on this issue.

### 3.2. Liver

Alterations in liver function are frequently observed in insulin resistance and type 2 diabetes mellitus. In fact, many patients suffering these metabolic alterations present nonalcoholic fatty liver disease (NAFLD), defined by a hepatic lipid accumulation >5% of the liver weight [60]. Hepatic lipid accumulation can also trigger inflammation, resulting in more severe liver disorders such as nonalcoholic steatohepatitis (NASH), cirrhosis, and hepatocellular carcinoma (HCC). Intriguingly, although hepatic lipid accumulation results from insulin resistance, it also contributes to hepatic insulin resistance [61], thereby suggesting that reversing hepatic steatosis can delay the progression from prediabetes to overt type 2 diabetes mellitus. Unregulated lipogenesis and reduced FA oxidation contribute to lipid accumulation in the liver, with AMPK regulating both processes in hepatocytes. Thus, as mentioned above, AMPK-mediated ACC inhibition leads to a decrease in intracellular levels of malonyl-CoA, which is both a precursor for FA biosynthesis and a potent allosteric inhibitor of FA oxidation. Moreover, AMPK reduces the expression of lipogenic genes by phosphorylating transcription factors such as sterol regulatory element binding protein-1c (SREBP-1c) [62] and carbohydrate-responsive element-binding protein (ChREBP) [63]. It has been reported that HFDs reduce hepatic phospho-AMPK levels and increase phospho-ERK levels, with GW501516 treatment preventing these changes by a mechanism that may involve an increased AMP/ATP ratio and elevated plasma β-hydroxybutyrate levels, indicating enhanced hepatic FA oxidation [64] (Figure 2). Interestingly, a different study reported that GW501516 treatment stimulated AMPK and ACC phosphorylation and attenuated FA synthesis in wild-type hepatocytes, but not in AMPKβ^-/-^ hepatocytes [65], thereby confirming the involvement of AMPK in these effects.

Autophagy is a catabolic process that delivers intracellular proteins and organelles to the lysosome during starvation for degradation and recycling, thereby promoting the redistribution of nutrients to maintain cellular energetic balance [66]. Notably, the inhibition of autophagy results in triglyceride accumulation and reduced FA oxidation in the liver, while drugs increasing autophagy alleviate liver steatosis in mice fed an HFD [67]. AMPK activation promotes autophagy through two different mechanisms: inhibition of the mammalian target of rapamycin (mTOR) protein kinase complex and direct phosphorylation of Unc-51-like kinase 1 (ULK1) [68]. Recently, it has been demonstrated that PPARβ/δ reduces hepatic steatosis and stimulates FA oxidation in the liver and hepatic cells by an autophagy-lysosomal pathway involving the AMPK-mTOR pathway [69] (Figure 2). More generally, the roles of PPARs and their novel ligands as potential drugs for the treatment of NAFLD have been reviewed recently [70].

Insulin resistance and type 2 diabetes mellitus are closely associated with a chronic low-grade inflammation characterized by an abnormal production of cytokines. Of these cytokines, interleukin 6 (IL-6) has been reported to induce hepatic insulin resistance [71]. IL-6 induces insulin resistance in the liver through the activation of signal transducer and activator of transcription 3 (STAT3) and the subsequent induction of suppressor of cytokine signaling 3 (SOCS3), which inhibits insulin signaling by interfering with insulin receptor activation, blocking IRS activation, and inducing IRS degradation [72]. In liver cells, PPARβ/δ activation was demonstrated to prevent IL-6-induced STAT3 activation and SOCS3 upregulation by counteracting the reduction in phospho-AMPK levels, which inhibits STAT3 phosphorylation [73] (Figure 2). Consistent with this, the livers of *Ppard*^−/−^ mice show increased phospho-STAT3 levels. This action of PPARβ/δ prevents the reduction in IRS-1 and IRS-2 levels caused by exposure of hepatic cells to IL-6 [73].

### 3.3. Heart

The risk of developing heart failure is higher in patients with insulin resistance and type 2 diabetes mellitus, with inflammation being a key systemic factor contributing to this relationship [74]. Indeed, the progression of cardiac hypertrophy and heart failure usually entails a local rise in proinflammatory factors, which are under the transcriptional control of nuclear factor-κB (NF-κB). Notably, AMPK activation may block NF-κB signaling through suppressing IκB kinase activity [75]. It has been reported that PPARβ/δ activation reduces the lipid-induced expression of NF-κB-target genes in the hearts of mice and in human cardiac cells, with these effects involving an AMPK-dependent mechanism [76] (Figure 2). In addition, NF-κB activity has been reported to be increased in the hearts of PPARβ/δ-knockout mice compared with wild-type mice, which is consistent with the anti-inflammatory effects of PPARβ/δ activity.

ER stress contributes to the pathogenesis of diabetic cardiomyopathy by promoting apoptotic cell death in the myocardium [77]. PPARβ/δ activation prevents lipid-induced ER stress in the heart by inducing autophagy [78]. In addition, PPARβ/δ-knockout mice display a reduction in autophagic markers. However, in contrast to what has been reported for the liver [69], these effects of PPARβ/δ occur in an AMPK-independent manner.

## 4. Going the Other Way: The AMPK-PPARβ/δ Pathway

**Figure 4:**
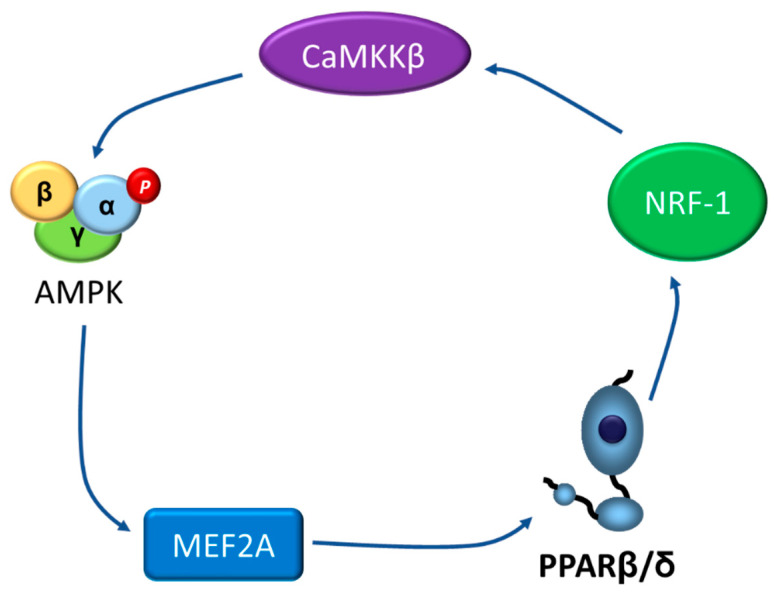
Potential positive loop between activated AMPK, PPARβ/δ, and MEF2A. AMPK, AMP-activated protein kinase; CaMKKβ, Ca^2+^/calmodulin-dependent protein kinase kinase-β; MEF2A, myocyte enhancer factor 2A; NRF-1: nuclear respiratory factor 1; PPARβ/δ, peroxisome proliferator-activated receptor β/δ.

While previous sections of this review clearly demonstrate that many of the antidiabetic effects of PPARβ/δ agonists are mediated via AMPK activation, a few studies have reported the opposite, i.e., the regulation of PPARβ/δ by AMPK. In fact, a recent study proposed the existence of a positive loop between activated AMPK, PPARβ/δ, and myocyte enhancer factor 2A (MEF2A), the latter being a transcription factor that upregulates the expression of *Ppard* and *Glut4* [79]. The authors of this study demonstrated that AMPK activation increases PPARβ/δ levels via MEF2A [79]. As mentioned above, increased levels of PPARβ/δ would activate the NRF-1/CaMKKβ pathway, thereby leading to AMPK activation, ultimately closing the loop (Figure 4). Thus, PPARβ/δ activates AMPK and AMPK activity influences PPARβ/δ levels, establishing a mutual cooperation that regulates MEF2A promoter activity and *Glut4* expression.

More recently, it has been reported that AMPK regulates PPARβ/δ phosphorylation, modulating its activity [80]. The authors of this study observed that the AMPK agonist metformin induced the phosphorylation of PPARβ/δ at Ser^50^ through the common LXRXXSXXXL phosphorylation motif recognized by this kinase, which localizes in the short N-terminal A/B activation domain of this nuclear receptor. Of note, AMPK-mediated phosphorylation of PPARβ/δ at Ser^50^ resulted in an accumulation of the protein levels of this PPAR isotype, suggesting that its phosphorylation attenuated PPARβ/δ degradation. In fact, PPARβ/δ phosphorylation at Ser^50^ inhibits the p62-mediated misfolded PPARβ/δ autophagic degradation. Despite the increase in PPARβ/δ levels caused by AMPK activation, the findings of this study suggest that PPARβ/δ phosphorylation inhibits transcriptional activity as a PPARβ/δ-Ser^50^ mutant showed increased activity compared with wild-type PPARβ/δ. Although this study was conducted in cancer cell lines, the AMPK-mediated phosphorylation of PPARβ/δ attenuated glucose uptake by reducing the expression of *Glut1*, thereby suggesting that this pathway can have metabolic implications. Further studies are needed to confirm whether this pathway operates in metabolic tissues such as the liver and skeletal muscle and how it regulates metabolism.

## 5. Conclusions and Perspectives

The development of novel drugs to treat type 2 diabetes mellitus continues to attract attention in the metabolism field. The PPARβ/δ-AMPK pathway is in the spotlight as it pharmacologically promotes the effects of exercise in skeletal muscle, such as increased glucose uptake and FA oxidation. This pathway also prevents lipid-induced ER stress and inflammation, thereby ameliorating insulin resistance. New specific molecular mechanisms indicating how this pathway ameliorates insulin resistance are beginning to emerge, such as the recently reported upregulation of GDF15 by PPARβ/δ agonists via AMPK. GDF15 upregulation activates AMPK, thereby implying that this mechanism contributes to the effects of PPARβ/δ agonists by sustaining AMPK activation. In addition, *Gdf15*^-/-^ mice show reduced AMPK activation in skeletal muscle, whereas GDF15 administration results in AMPK activation in this organ. Interestingly, this effect of GDF15 in AMPK activation seems to be independent of the central receptor GFRAL, thereby suggesting that this cytokine exerts autocrine/paracrine effects through yet to be determined receptors. Future studies aimed at expanding the mechanisms of action of the PPARβ/δ-AMPK pathway may facilitate the development of new antidiabetic compounds with improved efficacy and minimal side effects for the treatment of insulin resistance and the prevention of its progression to type 2 diabetes mellitus. In fact, type 2 diabetic patients might benefit from the development of new antidiabetic drugs targeting both PPARβ/δ and AMPK given the positive feedback loop that potentiates them each other. This might result in a new generation of molecules for the prevention and treatment of obesity-induced insulin resistance and type 2 diabetes mellitus. It is noteworthy that PPARβ/δ, similar to PPARα and PPARγ, has been ascribed pro- and anti-tumor activities that have to be considered in the development of new candidate drugs [5,81,82]. Several factors can contribute to the highly debated functional role of PPARβ/δ in tumorigenesis or carcinogenesis. For instance, the tumor promoter effects of PPARβ/δ agonists have been mostly observed in animal models. Although these animal models are a valuable tool for basic tumor research, they show some limitations and the conclusions obtained from these studies are not always confirmed in human beings. Thus, the expression of the different PPAR isotypes is higher in rodent than in human cells and the regulation of these nuclear receptors is also different depending on the cell type studied [5]. These differences may explain why, after decades of treating patients with the PPARα activators fibrates, no incidence of carcinogenesis has been reported, whereas it is well-known that administration of these drugs to rodents leads to carcinogenesis. Either way, as controversy about the role of PPARβ/δ agonists in cancer still remains, to minimize side effects, the success of PPARβ/δ-based treatment of insulin resistance would benefit from the development of innovative strategies for organ- or cell-type-specific drug delivery or release systems.

## Data Availability

Not applicable.
